# Time-dependent association of glucocorticoids with adverse outcome in community-acquired pneumonia: a 6-year prospective cohort study

**DOI:** 10.1186/s13054-017-1656-7

**Published:** 2017-03-24

**Authors:** Manuela Nickler, Manuel Ottiger, Christian Steuer, Alexander Kutz, Mirjam Christ-Crain, Werner Zimmerli, Robert Thomann, Claus Hoess, Christoph Henzen, Luca Bernasconi, Andreas Huber, Beat Mueller, Philipp Schuetz, for the ProHOSP Study Group, Ursula Schild, Katharina Regez, Rita Bossart, Robert Thomann, Claudine Falconnier, Marcel Wolbers, Stefanie Neidert, Thomas Fricker, Claudine Blum, Thomas Bregenzer, Claus Hoess, Heiner C. Bucher, Fabian Mueller, Jeannine Haeuptle, Roya Zarbosky, Rico Fiumefreddo, Melanie Wieland, Charly Nusbaumer, Andres Christ, Roland Bingisser, Kristian Schneider, Brigitte Walz, Verena Briner, Dieter Conen, Andreas Huber, Jody Staehelin, Chantal Bruehlhardt, Ruth Luginbuehl, Agnes Muehlemann, Ineke Lambinon, Werner Zimmerli, Max Zueger

**Affiliations:** 1 Medical University Department, Division of General Internal and Emergency Medicine, Kantonsspital Aarau, Aarau, Switzerland; 2 Department of Laboratory Medicine, Kantonsspital Aarau, Aarau, Switzerland; 3 Division of Endocrinology, Diabetes and Clinical Nutrition, Department of Internal Medicine, University Hospital Basel, Basel, Switzerland; 4 Medical Faculty, University of Basel, Basel, Switzerland; 5 Basel University Medical Clinic Liestal, Liestal, Switzerland; 6 Department of Internal Medicine, Bürgerspital Solothurn, Solothurn, Switzerland; 7 Department of Internal Medicine, Kantonsspital Münsterlingen, Münsterlingen, Switzerland; 8 Department of Internal Medicine, Kantonsspital Lucerne, Lucerne, Switzerland

**Keywords:** Community-acquired pneumonia, Glucocorticoid hormones, Cortisol, 11-Deoxycortisol, Cortisone, Corticosterone, Mortality/outcome prediction, Disease severity, Pneumonia severity index

## Abstract

**Background:**

The hypothalamic-pituitary-adrenal stress axis plays a crucial role in community-acquired pneumonia (CAP), with high cortisol being associated with disease severity and corticosteroid treatment resulting in earlier time to recovery. Our aim in the present study was to compare different glucocorticoid hormones, including cortisol, 11-deoxycortisol, cortisone, and corticosterone, regarding their association with short- and long-term adverse outcomes in a well-defined CAP cohort.

**Methods:**

We prospectively followed 285 patients with CAP from a previous Swiss multicenter trial for a median of 6.1 years and measured different admission glucocorticoid serum levels by liquid chromatography coupled with tandem mass spectrometry. We used adjusted Cox regression models to investigate associations between admission hormone levels and all-cause mortality at different time points.

**Results:**

Mortality was 5.3% after 30 days and increased to 47.3% after 6 years. High admission cortisol was associated with adverse outcome after 30 days (adjusted OR 3.85, 95% CI 1.10–13.49, *p* = 0.035). In the long term (i.e.,), however, high admission cortisol was associated with better survival (adjusted HR after 3 years 0.53, 95% CI 0.32–0.89, *p* = 0.017; adjusted HR after 6 years 0.57, 95% CI 0.36–0.90, *p* = 0.015). Compared with 11-deoxycortisol, cortisone, and corticosterone, cortisol showed the highest association with mortality.

**Conclusions:**

Among different glucocorticoid hormones, cortisol showed the highest association with mortality in CAP. Whereas a more pronounced glucocorticoid stress response on hospital admission was associated with higher short-term adverse outcome, long-term outcome was favorable in these patients. These data should support the correct interpretation of glucocorticoid blood data.

**Electronic supplementary material:**

The online version of this article (doi:10.1186/s13054-017-1656-7) contains supplementary material, which is available to authorized users.

## Background

Activation of the hypothalamic-pituitary-adrenal (HPA) axis and stimulation of the central noradrenergic stress system by cytokines and other mediators are major pathophysiological adaptations in response to infection and inflammation [1–4]. Appropriate activation of the HPA axis during illness is crucial for survival and mirrors the stress level. The effects of cortisol include protection against excessive inflammatory reaction, acute supply of energy, and improvement in hemodynamic status [5, 6].

Endogenous adrenocortical function is of prognostic value in critical illness and sepsis [7–14], of which community-acquired pneumonia (CAP) is a major source [15–17]. Morbidity in CAP is still high also, owing to higher risk for cardiovascular events [18, 19]. Researchers in multiple studies have investigated the adrenal steroid metabolism in relation to CAP-associated outcomes [14, 20–25]. In observational trials, researchers have reported increased admission cortisol levels to be independent predictors of disease severity and short-term mortality in patients across the spectrum of mild to severe CAP [20–24]. Interestingly, the predictive accuracy of free cortisol was not superior to that of total cortisol, independent of serum albumin levels [20]. The prognostic accuracy of cortisol levels in short-term mortality prediction was equal to that of the pneumonia severity index (PSI), but better than the CURB-65 score (confusion of new onset, blood urea nitrogen >7 mmol/L, respiratory rate ≥30 breaths per minute, systolic blood pressure <90 mmHg or diastolic blood pressure ≤60, and age ≥65 years) and routinely measured laboratory parameters such as C-reactive protein (CRP), procalcitonin (PCT), or white blood cell count [20, 21, 23]. In addition, in severe CAP, baseline serum cortisol levels were shown to be independent predictors of mortality with superior predictive ability compared with stimulated cortisol with corticotropin or Δ-cortisol [24]. Despite evidence regarding short-term outcomes, less is known about the predictive potential of glucocorticoid levels in regard to long-term mortality. Aside from that, most studies have been focused on cortisol only, and data regarding other human glucocorticoids, such as 11-deoxycortisol, cortisone, and corticosterone, are largely lacking. Identification of patients at risk for adverse outcome after an index hospitalization for CAP may optimize in-hospital and post-acute care strategies to improve survival. A better description of metabolite signatures in CAP may improve pathophysiological understanding and provide new targets for a more personalized therapy. In the present study, our aim was to study different compounds of the glucocorticoid pathway, including cortisol, 11-deoxycortisol, cortisone, and corticosterone, regarding their association with short- and long-term adverse outcomes in a well-defined CAP cohort.

## Methods

### Study design and setting

This is a secondary analysis of data from a prospective, randomized, controlled multicenter trial performed at six Swiss secondary or tertiary care centers between October 2006 and March 2008 [26]. A detailed study protocol of the initial trial has been published elsewhere [27]. Briefly, from a total of 1825 potential patients, 925 subjects with CAP were included. The aim of the initial trial was to assess the efficacy and safety of a PCT-guided antibiotic therapy compared with standard guidelines without using PCT data [28–30] in patients with CAP and other lower respiratory tract infections [31]. The study protocol was approved by the ethics committee of the University of Basel as well as by all local ethics committees, and written informed consent was provided by all participants for the initial trial, including agreement to use their data anonymized in secondary analyses.

### Selection and assessment of participants

Inclusion criteria were age >18 years and final diagnosis of CAP with an infiltrate on a chest x-ray [31]. Exclusion criteria were language restriction or dementia precluding patients from providing written informed consent, presence of a terminal condition, and intravenous drug abuse. For reasons of the initial antibiotic stewardship trial, patients were excluded for severe immunosuppression or for long-term antibiotic therapy on admission independent of the current infection (e.g., in case of endocarditis or chronic infections), whereas a requirement of corticosteroids or short-term antibiotic pretreatment was allowed. All patients included in the analysis were inpatients.

Patient assessment included clinical and biochemical evaluation upon admission to the emergency department (ED) and throughout the period of hospitalization. The standardized baseline characteristics comprised medical history, comorbidities (identified either through patient self-report or medical chart review), vital signs including fever (≥38.5 °C), laboratory values, chest x-ray, and medication. To assess disease severity, the PSI [32] and the CURB-65 score [29]—validated risk assessment tools to categorize patients with CAP into different risk classes—were calculated upon ED admission. Confusion was also assessed as part of these scores. Discharge decision concerning patients enrolled in the study was left completely to the treating physicians without interference of the study team. For the present study, 285 patients with a final diagnosis of CAP and available serum blood specimens [27, 31] were included.

### Analysis of blood biomarker

Within the initial trial, blood samples from each patient were collected and frozen upon ED admission for later measurement of different biomarkers. Cortisol, 11-deoxycortisol, cortisone, and corticosterone levels were measured in admission serum blood samples of all 285 included patients with CAP.

After internal validation studies, we determined concentrations of selected hormones using a commercially available kit (MassChrom Steroids; Chromsystems, Munich, Germany). The analysis was performed using the UltiMate 3000 ultra-high-performance liquid chromatography (UHPLC) system (Thermo Fisher Scientific, San Jose, CA, USA) coupled to an AB Sciex 5500 quadrupole mass spectrometer (AB Sciex, Darmstadt, Germany). The Turbo V ion source (AB Sciex) was operated in positive electrospray ionization mode. The targeted screening method employed the multiple reaction monitoring mode of operation using two transitions for each analysis sample. Prior to injection into the UHPLC system, serum samples were subjected to a complex process of reversed phase 96-well solid-phase extraction, purification, and concentration as described in the MassChrom Steroids user’s manual. Quantification of selected metabolites was achieved by reference to appropriate internal standards. Concentrations of all analyzed metabolites were reported in nanomoles per liter.

### Main outcome measurements

The primary endpoint of this study was defined as 6-year all-cause mortality. As secondary endpoints, we reported mortality at days 30, 60, 90, 180, 240, and 300, as well as at 1 year, 2 years, and 3 years. Additional secondary endpoints were 30-day adverse outcome (including all-cause mortality and/or admission to the intensive care unit [ICU]) and disease severity defined by the PSI on admission. The decision for ICU admission was left to the treating physicians.

To validate outcomes, we performed blinded, structured telephone interviews at 30, 180, and 540 days after enrollment, as well as after a median of 6.1 years (IQR 5.6–6.5) [33, 34]. In cases where patients or their family members could not be contacted, the treating general practitioner was contacted.

### Statistical analyses

Statistical analyses were conducted using STATA 12.1 software (StataCorp, College Station, TX, USA). *p* Values <0.05 indicated statistical significance. Description of the study population was performed with descriptive statistics, including median with IQR to express continuous variables and frequency (percent) for categorical variables. The Wilcoxon rank-sum test was used for two-group comparison, whereas frequency comparison was done by chi-square test. All analyses were performed in the overall population.

Correlation analyses of glucocorticoid levels with inflammatory markers were calculated by Spearman’s rank correlation. For multigroup comparisons, the Kruskal-Wallis test was performed. Univariate and multivariate Cox regression models were performed to investigate associations between glucocorticoid levels and all-cause mortality at different time points; these associations were reported as HR with 95% CI. Before being entered into regression models, blood biomarker levels were log-transformed with a base of 10 due to a skewed distribution; after logarithmic transformation, the data approximated a normal distribution. Because of this transformation, HRs and ORs correspond to a tenfold increase in hormone levels. Kaplan-Meier curves were used to illustrate mortality based on glucocorticoid quartiles (highest versus lower three). To assess the association of glucocorticoid levels with other short-term adverse outcomes, we used univariate and multivariate logistic regression models, reported as OR with 95% CI. Significance levels were calculated by chi-square (Wald) test.

Models were adjusted for age, sex, and comorbidities (coronary heart disease, cerebrovascular insult, chronic kidney disease, neoplastic disease). The multivariate adjustment was predefined on the basis of factors known to be associated with mortality (age and comorbidities). We also included sex in the model because of expected differences in adrenal hormone levels based on gender. Initial randomization was not significantly associated with study endpoints and was thus not further considered for the statistical analysis.

## Results

### Patient population

Among a total of 285 patients with CAP, 135 (47.3%) died during the 6-year follow-up period. The median age of the entire cohort was 71 years, and 60.4% of patients were male. There was a high burden of comorbidities, with 20.7% (*n* = 59) of patients having underlying coronary heart disease, 15.4% (*n* = 44) having congestive heart failure, 23.5% (*n* = 67) having chronic renal failure, 19.3% (*n* = 55) having diabetes mellitus, and 13.3% (*n* = 38) having neoplastic disease. We had blood levels available for cortisol in 227 patients (80%), for 11-deoxycortisol in 249 patients (87%), for cortisone in 283 patients (99%), and for corticosterone in 285 patients (100%). Additional baseline characteristics of the entire cohort, stratified by the primary endpoint as well as by 30-day adverse outcome, are shown in Table 1 and Additional file 1: Table S1, respectively.

**Table 1:** Baseline characteristics overall and stratified by 6-year vital status in community-acquired pneumonia

		6-Year vital status		
Characteristics	Entire cohort (*n* = 285)	Survivors (*n* = 150)	Nonsurvivors (*n* = 135)	*p* Value
Demographic characteristics				
Age, years	71 [57–81]	64 [45–75]	79 [70–84]	**<0.001**
Male sex	172 (60.4%)	80 (53.3%)	92 (68.1%)	**0.011**
CAP characteristics				
PSI class				
I	32 (11.2%)	30 (20.0%)	2 (1.5%)	**<0.001**
II	55 (19.3%)	44 (29.3%)	11 (8.1%)	**<0.001**
III	52 (18.2%)	29 (19.3%)	23 (17.0%)	0.62
IV	104 (36.5%)	38 (25.3%)	66 (48.9%)	**<0.001**
V	42 (14.7%)	9 (6.0%)	33 (24.4%)	**<0.001**
CURB-65 score				
0	63 (22.1%)	51 (34.0%)	12 (8.9%)	**<0.001**
I	67 (23.5%)	41 (27.3%)	26 (19.3%)	0.11
II	82 (28.8%)	33 (22.0%)	49 (36.3%)	**0.008**
III	57 (20.0%)	21 (14.0%)	36 (26.7%)	**0.008**
IV/V	16 (5.6%)	4 (2.7%)	12 (8.9%)	**0.023**
Comorbidities^a^				
Coronary heart disease	59 (20.7%)	16 (10.7%)	43 (31.9%)	**<0.001**
Congestive heart failure	44 (15.4%)	7 (4.7%)	37 (27.4%)	**<0.001**
Cerebrovascular insult	28 (9.8%)	9 (6.0%)	19 (14.1%)	**0.022**
PAOD	17 (6.0%)	7 (4.7%)	10 (7.4%)	0.33
Chronic renal failure	67 (23.5%)	19 (12.7%)	48 (35.6%)	**<0.001**
Diabetes mellitus	55 (19.3%)	22 (14.7%)	33 (24.4%)	**0.037**
Neoplastic disease	38 (13.3%)	12 (8.0%)	26 (19.3%)	**0.005**
Clinical history				
Fever	185 (65.1%)	113 (75.3%)	72 (53.7%)	**<0.001**
Chills	87 (34.0%)	58 (42.0%)	29 (24.6%)	**0.003**
Glucocorticoid pretreatment	22 (7.9%)	5 (3.4%)	17 (12.9%)	**0.003**
Clinical findings				
Confusion	20 (7.9%)	4 (2.9%)	16 (13.7%)	**0.002**
Body temperature, °C	38 [37.2–38.8]	38.2 [37.4–39]	37.8 [37–38.8]	0.085
Breath rate, breaths/minute	20 [16–25]	20 [16–24]	24 [18–28]	**0.002**
Heart rate, beats/minute	94 [82–105]	92.5 [83.5–108]	95 [80–104]	0.43
SBP, mmHg	130 [117–148]	130 [120–148]	130 [110–149]	0.17
Arterial pH	7.46 [7.42–7.49]	7.46 [7.43–7.50]	7.45 [7.41–7.49]	**0.013**
SIRS criteria	188 (66.0%)	93 (62.0%)	95 (70.4%)	0.14
Outcome parameters				
ICU admission	21 (7.4%)	9 (6.0%)	12 (8.9%)	0.35
Mechanical ventilation	7 (2.5%)	2 (1.3%)	5 (3.7%)	0.20
Septic shock	6 (2.1%)	1 (0.7%)	5 (3.7%)	0.075
Length of stay, days	8 [5–12]	7 [4–10]	9 [6–13]	**<0.001**
Admission laboratory findings				
CRP, mg/L	132 [65–252]	147 [91–265]	111 [55–249]	**0.037**
PCT, μg/L	0.48 [0.16–3.20]	0.64 [0.16–3.72]	0.43 [0.16–1.97]	0.37
Cortisol, nmol/L	402 [203.8–723.2]	431 [189.1–759.2]	399 [213.1–710.3]	0.46
11-Deoxycortisol, nmol/L	0.6 [0.17–2.23]	0.4 [0.16–2.13]	0.8 [0.18–2.25]	0.49
Cortisone, nmol/L	32.5 [18.42–46.72]	33.2 [19.28–48.49]	32.4 [17.68–44.41]	0.29
Corticosterone, nmol/L	8.7 [2.81–25.02]	8.9 [2.73–28.67]	8.4 [3.13–22.95]	0.85

*Abbreviations: CAP* Community-acquired pneumonia, *CRP* C-reactive protein, *CURB-65* Confusion of new onset, blood urea nitrogen >7 mmol/L, respiratory rate ≥30 breaths per minute, systolic blood pressure <90 mmHg or diastolic blood pressure ≤60, and age ≥65 years, *PAOD* Peripheral arterial occlusive disease, *PCT* Procalcitonin, *PSI* Pneumonia severity index, *SBP* Systolic blood pressure, *SIRS* Systemic inflammatory response syndrome, *ICU* Intensive care unit

Data are presented as median [IQR] or number (percent); *p* < 0.05 is considered statistically significant. Bold values indicate statistical significance

^a^Comorbidities were identified on the basis of medical records or patient report

Importantly, compared with the study population of the initial trial [26], including 925 patients with CAP with an all-cause mortality of 45% over a follow-up period of 6 years, the present analyzed cohort displayed a similar pattern of baseline characteristics [34]. Mortality in this cohort was 5.3% after 30 days and increased to 20.7% and 47.3% after 3 years and 6 years, respectively.

### Time-dependent association between glucocorticoid levels and adverse outcome

**Fig. 1:**
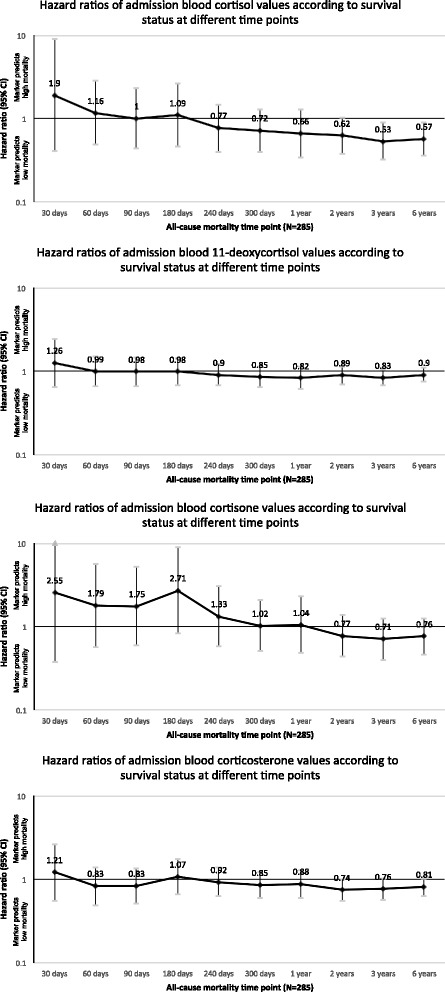
Association of admission glucocorticoid levels with all-cause mortality at different time points in CAP. Data for multivariate Cox regression models are presented as HR (95% CI). HRs >1 reflect a positive association between glucocorticoid levels and all-cause mortality. Multivariate model is adjusted for age, sex, and comorbidities (coronary artery disease, cerebrovascular disease, chronic kidney disease, neoplastic disease). *CAP* Community-acquired pneumonia

**Fig. 2:**
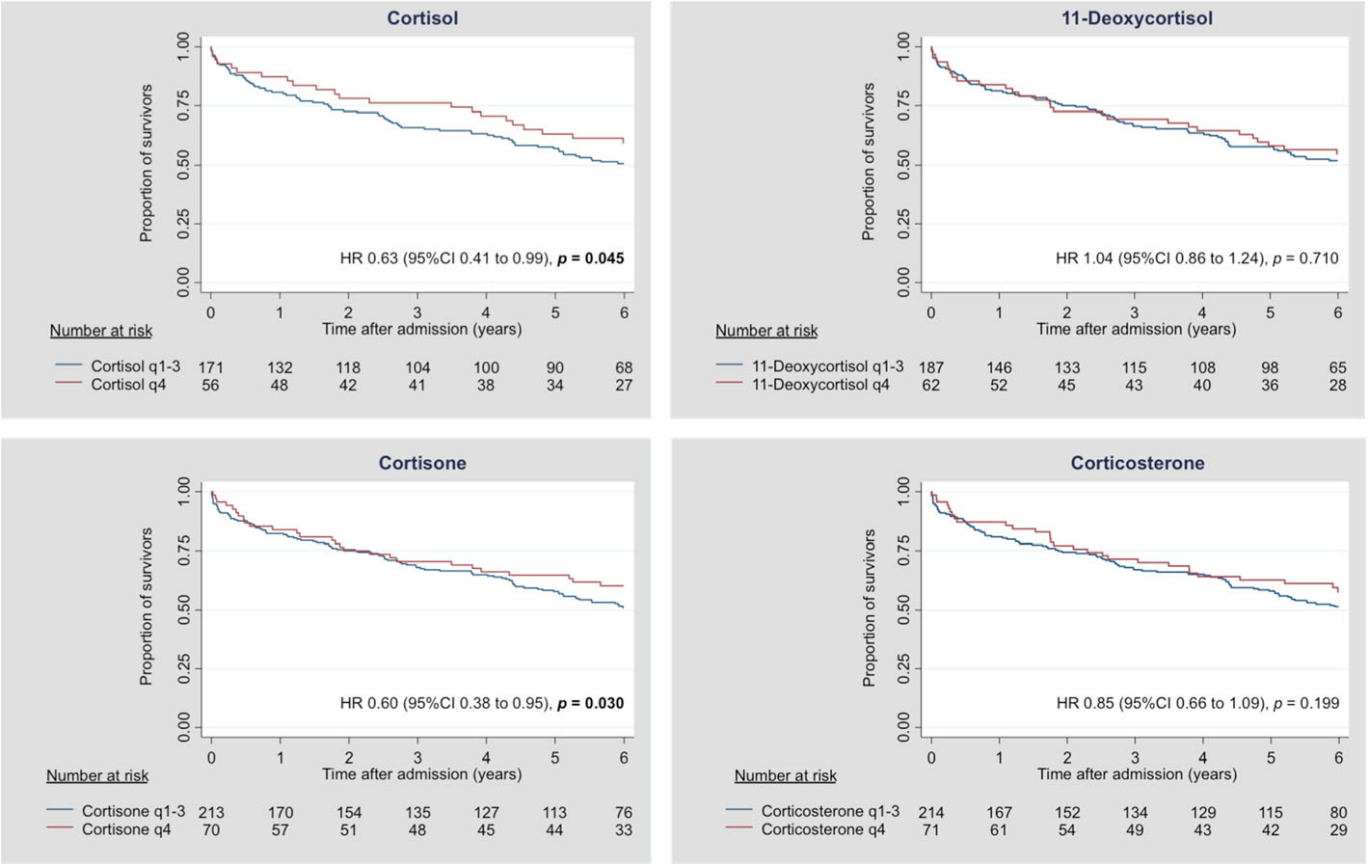
Kaplan-Meier 6-year survival estimate according to admission glucocorticoid levels in CAP: fourth versus first-to-third quartiles. *CAP* Community-acquired pneumonia

Table 2 illustrates associations between admission serum glucocorticoid levels and all-cause mortality in a time-dependent manner. In the short term, all glucocorticoid hormones had HRs >1 and thus tended to be associated with higher mortality risk, but these associations changed over time. We found high initial cortisol and cortisone levels to be significantly associated with lower risk for 6-year mortality with HRs of 0.63 (95% CI 0.41–0.99; *p* = 0.045) and 0.60 (95% CI 0.38–0.95; *p* = 0.030), respectively. For 3-year mortality, results were similar with HRs of 0.54 (95% CI 0.32–0.90; *p* = 0.018) and 0.56 (95% CI 0.33–0.94; *p* = 0.028) for cortisol and cortisone, respectively. For cortisol, these associations remained robust after adjustment for age, sex, and comorbidities. These results are also presented in Fig. 1, where a stepwise decrease in HRs with longer observation time for cortisol and cortisone, and to a lesser extent also for 11-deoxycortisol and corticosterone, can be observed. We further show Kaplan-Meier curves for 6-year survival stratified by glucocorticoid-level quartiles (Fig. 2). The highest quartile of admission cortisol and cortisone levels was favorably associated with long-term survival.

**Table 2:** Association of admission glucocorticoid levels with short- and long-term all-cause mortality in community-acquired pneumonia

Entire cohort (*N* = 285)	All-cause mortality time point					
	30 days		3 years		6 years	
	HR (95% CI)	*p* Value	HR (95% CI)	*p* Value	HR (95% CI)	*p* Value
Cortisol						
Cox regression analyses						
Univariate model	2.47 (95% CI 0.50–12.18)	0.268	0.54 (95% CI 0.32–0.9)	**0.018**	0.63 (95% CI 0.41–0.99)	**0.045**
Multivariate model^a^	1.93 (95% CI 0.51–9.02)	0.405	0.53 (95% CI 0.32–0.89)	**0.017**	0.57 (95% CI 0.36–0.90)	**0.015**
11-Deoxycortisol						
Cox regression analyses						
Univariate model	1.37 (95% CI 0.75–2.53)	0.309	0.95 (95% CI 0.76–1.18)	0.631	1.04 (95% CI 0.86–1.24)	0.710
Multivariate model^a^	1.26 (95% CI 0.65–2.44)	0.486	0.84 (95% CI 0.67–1.05)	0.126	0.91 (95% CI 0.75–1.1)	0.336
Cortisone						
Cox regression analyses						
Univariate model	1.37 (95% CI 0.31–6.02)	0.677	0.56 (95% CI 0.33–0.94)	**0.028**	0.6 (95% CI 0.38–0.95)	**0.030**
Multivariate model^a^	2.55 (95% CI 0.38–17.30)	0.337	0.71 (95% CI 0.4–1.25)	0.237	0.76 (95% CI 0.46–1.24)	0.272
Corticosterone						
Cox regression analyses						
Univariate model	1.33 (95% CI 0.63–2.81)	0.457	0.77 (95% CI 0.57–1.03)	0.082	0.85 (95% CI 0.66–1.09)	0.199
Multivariate model^a^	1.21 (95% CI 0.55–2.65)	0.637	0.76 (95% CI 0.57–1.02)	0.067	0.81 (95% CI 0.63–1.03)	0.084

Data for univariate and multivariate Cox regression models are presented as HR (95% CI), *p* value; *p* < 0.05 is considered statistically significant. Bold values indicate statistical significance. All hormone levels were log-transformed, and thus the HR corresponds to a tenfold increase in these levels

^a^Multivariate model is adjusted for age, sex, and comorbidities (coronary artery disease, cerebrovascular disease, chronic kidney disease, neoplastic disease)

Also, high initial levels of cortisol were independently associated with higher risk for short-term adverse outcome (defined as risk for mortality and/or ICU admission) with an adjusted OR of 3.85 (95% CI 1.10–13.49; *p* = 0.035) (Table 3).

**Table 3:** Association of admission glucocorticoid levels with short-term adverse outcome in community-acquired pneumonia

Entire cohort (*N* = 285)	Adverse outcome at 30 days (death and/or ICU admission)	
	OR (95% CI)	*p* Value
Cortisol		
Logistic regression analyses		
Univariate model	4.16 (95% CI 1.22–14.12)	**0.022**
Multivariate model^a^	3.85 (95% CI 1.10–13.49)	**0.035**
11-Deoxycortisol		
Logistic regression analyses		
Univariate model	1.33 (95% CI 0.84–2.11)	0.216
Multivariate model^a^	1.30 (95% CI 0.80–2.14)	0.293
Cortisone		
Logistic regression analyses		
Univariate model	1.89 (95% CI 0.59–1.03)	0.280
Multivariate model^a^	3.25 (95% CI 0.84–12.68)	0.089
Corticosterone		
Logistic regression analyses		
Univariate model	1.41 (95% CI 0.81–2.47)	0.225
Multivariate model^a^	1.44 (95% CI 0.79–2.62)	0.234

*ICU*, Intensive care unit

Data for univariate and multivariate logistic regression models are presented as OR (95% CI), *p* value; *p* < 0.05 is considered statistically significant. Bold values indicate statistical significance. All hormone levels were log-transformed, and thus the OR corresponds to a tenfold increase in levels

^a^Multivariate model is adjusted for age, sex, and comorbidities (coronary artery disease, cerebrovascular disease, chronic kidney disease, neoplastic disease)

### Association between glucocorticoid levels and severity of CAP

**Fig. 3:**
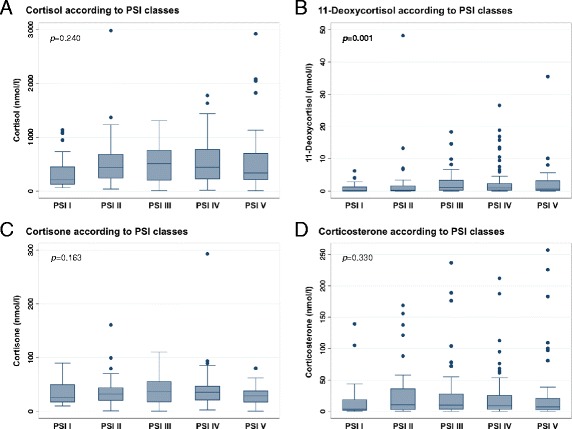
Glucocorticoid levels in patients with various severity classes of CAP. Data represent median and IQR, with scatter plots presenting all values. *P* values are determined by Kruskal-Wallis test and considered statistically significant at *p* < 0.05. Bold values indicate statistical significance. *CAP* Community-acquired pneumonia, *PSI* Pneumonia severity index

Multigroup comparison using the Kruskal-Wallis test showed a gradual increase in initial 11-deoxycortisol levels with increasing CAP severity as assessed by PSI classes (*p* = 0.001) (illustrated in Fig. 3). Contrarily, there was no similar increase in levels of cortisol, cortisone, and corticosterone with PSI. However, for cortisol, there was a trend of increasing hormone levels in more severely ill patients.

### Correlation between glucocorticoid levels and inflammatory markers

**Fig. 4:**
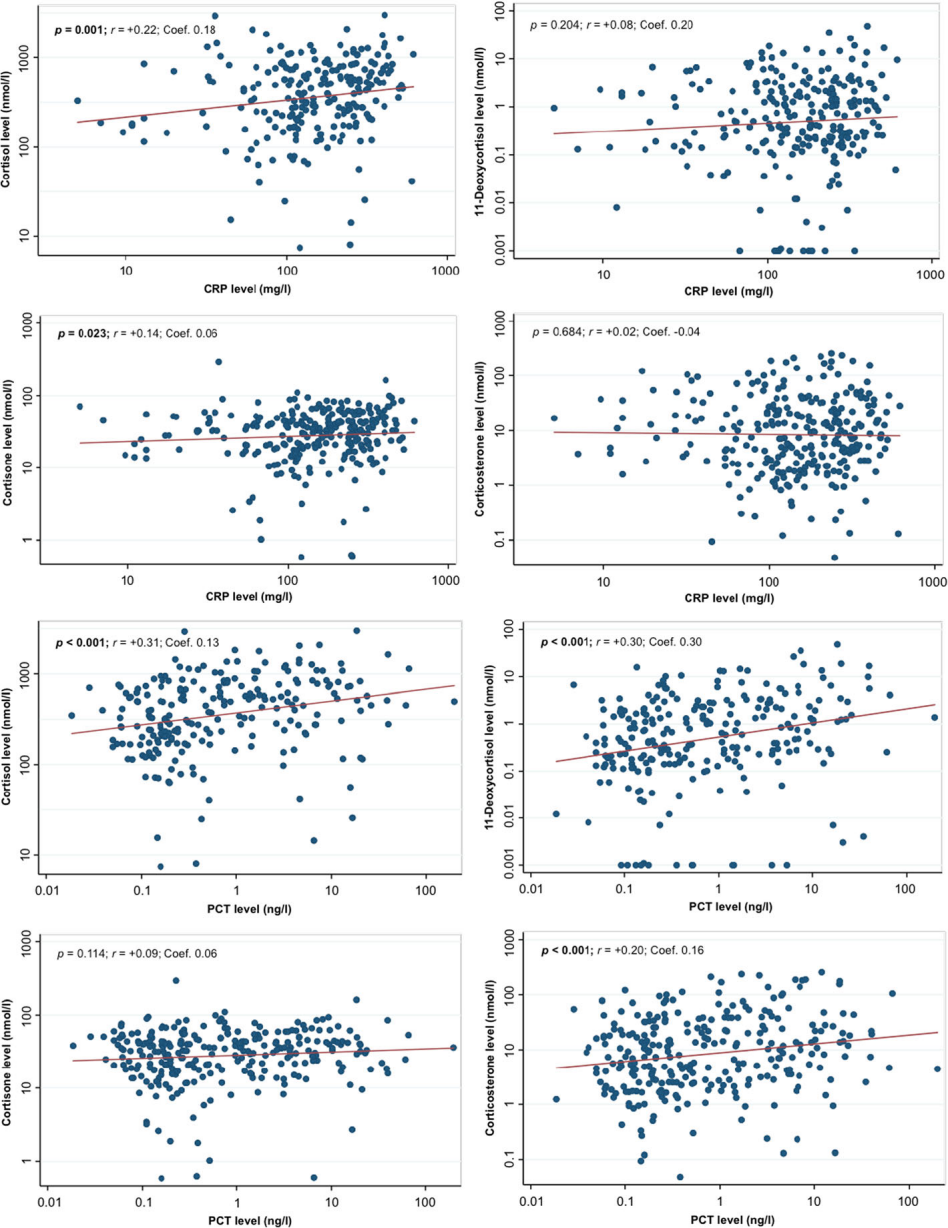
Correlation of admission glucocorticoid levels with inflammatory markers in CAP. Data are presented with scatterplots showing all values (*blue*), overlaid by linear fit lines (*red*). Correlation analyses were performed by Spearman’s rank correlation (*r*; *p* value). We used multivariate linear regression models to calculate regression coefficients (Coef.). *p* < 0.05 is considered statistically significant. Bold values indicate statistical significance. We used admission glucocorticoid and PCT levels and high peak CRP levels. *CAP* Community-acquired pneumonia, *CRP* C-reactive protein, *PCT* Procalcitonin. *Multivariate model is adjusted for age, sex, and comorbidities (coronary artery disease, cerebrovascular disease, chronic kidney disease, neoplastic disease)

As presented in Fig. 4, we found a correlation between admission cortisol levels and high peak CRP (*r* = +0.22; *p* = 0.001) and initial PCT values (*r* = +0.31; *p* < 0.001). Cortisone levels were also positively correlated with CRP levels (*r* = +0.14; *p* = 0.023), whereas 11-deoxycortisol and corticosterone were significantly correlated with PCT levels (*r* = +0.30 and +0.20, respectively; *p* < 0.001 for both).

## Discussion

The key findings of our present study of the prognostic value of different glucocorticoid hormones over a follow-up period of 6 years in patients with CAP are threefold. First, among different glucocorticoid hormones, cortisol showed the highest association with outcome in the short and long term. Second, the association of different glucocorticoid metabolites with adverse outcome inversely changed over time; whereas high levels of cortisol on initial hospital presentation were independently associated with short-term adverse outcome, a more pronounced initial glucocorticoid stress response was associated with favorable long-term outcome. Third, admission levels of 11-deoxycortisol showed the best correlation with disease severity.

The HPA stress axis plays an important role in patients with CAP, with cortisol being associated with short-term outcome [20–24] and corticosteroid treatment that might result in earlier time to recovery [35]. The release of cortisol in acute illness is essential for improvement in hemodynamic status, acute supply of energy, and protection against excessive inflammatory reaction [5, 6]. Importantly, the acute stress response involves activation of the glucocorticoid pathway and the adrenergic system, resulting in tachycardia, increased myocardial oxygen consumption, and probably enhanced platelet aggregation, which might translate into acute myocardial infarction, thus potentially raising short-term mortality [18]. Owing to its positive correlation with the degree of illness-induced stress [36], cortisol has been presumed to be a prognostic marker mirroring characteristics of acute critical disease and disease progression. Considering cortisol as the strongest inhibitor of inflammation, a higher level of proinflammatory cytokines in acute illness could lead to a more pronounced increase in cortisol [1–3, 37]. On the basis of several clinical observational studies [20–24], cortisol levels have lately emerged as a useful prognostic tool regarding short-term mortality. The present analysis of a large, well-characterized cohort provides novel insights into the role of the glucocorticoid pathway in regard to adverse outcome in hospitalized patients with mild to severe CAP.

Regarding short-term outcome, our findings are in line with previous data derived from clinical observational studies suggesting that high levels of cortisol ae associated with mortality in CAP [20–24]. In our study, high admission cortisol levels were independently associated with short-term adverse outcome, including the combined endpoint mortality/ICU admission. Association of cortisol levels with 30-day all-cause mortality alone showed a similar trend; likely due to the small event number, the results did not reach statistical significance.

Interestingly, a more pronounced initial glucocorticoid host response was beneficial for long-term survival. For cortisol, this effect remained robust after adjustment for potential confounders. Because most previous studies have demonstrated a strong HPA stress axis response to be positively associated with adverse short-term outcomes [20–24], the present findings are somewhat counterintuitive. Hypothesizing that high levels of proinflammatory cytokines lead to an increase in cortisol [1–3, 37] to avoid excessive inflammation, a more pronounced initial glucocorticoid response might reflect not only the degree of stress but also the ability to generate an adequate immune response. Within this study, we demonstrated an independent correlation between admission cortisol levels and markers of inflammation (PCT and high peak CRP). From this point of view, our findings are in line with those of a previous study demonstrating a more pronounced inflammatory host response reflected by a history of chills, high body temperature, and high peak levels of CRP being associated with lower long-term mortality in patients surviving a CAP episode [38]. These results were similar to those of the Pneumonia Patient Outcomes Research Team cohort study, another large follow-up study of patients with CAP, in which researchers reported an association between the absence of fever and higher long-term mortality than among age-matched control subjects [39]. Contrarily, several studies have shown an association between worse short-term outcomes and strong inflammatory response in CAP [40–42]. On the basis of these data, it is tempting to hypothesize that survival of a clinically and biochemically pronounced CAP episode—reflected by high admission glucocorticoid levels—might mirror a more robust stress response, and, concordantly, also host defense, and thus a better general condition with lower mortality in the long term. However, a pronounced glucocorticoid response on admission might reflect, as a presumable consequence of inflammation, the ability to generate an appropriate immune response, thereby predisposing these “fitter” patients, with regard to the stress-response, to better long-term survival owing to decreased subsequent infections [43]. Further investigations are still warranted to validate these findings and determine underlying pathophysiology. Given the evidence of pronounced inflammation being associated with favorable long-term outcome in hospitalized patients with CAP, the rationale of steroid treatment in this population should be questioned.

The main strengths of this study include the well-characterized cohort of patients hospitalized for CAP, the long follow-up period of approximately 6 years, and the thorough and highly accurate biomarker measurement by liquid chromatography coupled with tandem mass spectrometry. This technique allows a proper separation and sensitive characterization of even small molecules [44]. Importantly, mass spectrometry is increasingly becoming accepted as a routine diagnostic instrument in clinical laboratories [45]. Furthermore, the large sample size and the high event number of the primary endpoint (47.3%) lead to high statistical power, which allows detecting even small differences in glucocorticoid levels.

The following limitations require consideration. First, the present study is a secondary analysis and therefore not designed primarily to perform observational biomarker outcome studies. Importantly, there was no controlling for the time point of blood sampling, because initial blood samples were taken at the time of first patient contact in the ED. However, although cortisol exhibits diurnal concentration changes, during acute illness, the circadian pattern is usually lost [5, 46, 47]. Also, the long storage of blood may have affected blood hormone levels. In addition, there was no assessment of adrenal insufficiency based on the response to injection of synthetic adrenocorticotropin. Nevertheless, previous data demonstrated a very low rate of adrenal insufficiency in patients with CAP in the absence of septic shock [23]. However, deviation of the present results by different blood sampling time points or adrenal insufficiency cannot be definitively ruled out, although this lack of standardization reflects daily clinical practice. A more standardized hormone level determination at the same time of the day in all patients would most probably have demonstrated an even higher association between glucocorticoid levels and CAP outcomes. Moreover, serial hormone measurements over the course of the disease may add valuable information.

Second, the initial trial was conducted in different secondary and tertiary care hospitals in Switzerland, and thus results may not unconditionally be applied to other geographical or institutional settings. In addition, our analysis was focused specifically on patients with CAP; further studies are required to validate these findings for other medical and surgical patient populations. Finally, because this was an observational study, it is only hypothesis-generating, and correlation does not imply a causal relationship.

## Conclusions

Cortisol showed the highest association with outcome in patients with mild to severe CAP. The association of the glucocorticoid levels with adverse outcome in patients with CAP changed over time; whereas high admission levels of cortisol were independently associated with short-term adverse outcome, a more pronounced initial glucocorticoid stress response was highly associated with favorable long-term outcome. Initial 11-deoxycortisol levels showed the highest correlation with disease severity. Underlying pathophysiological aspects are still poorly examined, and future studies are needed to better understand the importance of adrenal hormones in the resolution of patients with CAP.

## Key messages

- Among different glucocorticoid hormones, cortisol showed the highest association with mortality in patients with CAP.
- The association of glucocorticoid metabolites with outcome in CAP changed over time; whereas a more pronounced glucocorticoid stress response on initial hospital presentation was associated with higher short-term adverse outcome, increased admission hormone levels showed association with favorable long-term outcome.

## Additional file

Additional file 1: Table S1. Baseline characteristics overall and stratified by 30-day adverse outcome, including the combined endpoint death/ICU admission in CAP. Data are presented as median [IQR] or number (percent); *p* values are considered statistically significant at *p* < 0.05. Bold values indicate statistical significance. CAP, Community-acquired pneumonia; CRP, C-reactive protein; CURB65, Confusion of new onset, blood urea nitrogen >7 mmol/L, respiratory rate ≥30 breaths per minute, systolic blood pressure <90 mmHg or diastolic blood pressure ≤60, and age ≥65 years; ICU, Intensive care unit; PAOD, Peripheral arterial occlusive disease; PCT, Procalcitonin; PSI, Pneumonia severity index; SBP, Systolic blood pressure; SIRS, Systemic inflammatory response syndrome. *Comorbidities were identified on the basis of medical records or patient report. (DOCX 21 kb)

## Abbreviations

CAP: Community-acquired pneumonia
CRP: C-reactive protein
CURB-65: Confusion of new onset, blood urea nitrogen >7 mmol/L, respiratory rate ≥30 breaths per minute, systolic blood pressure <90 mmHg or diastolic blood pressure ≤60, and age ≥65 years
ED: Emergency department
HPA: Hypothalamic-pituitary-adrenal
ICU: Intensive care unit
PAOD: Peripheral arterial occlusive disease
PCT: Procalcitonin
PSI: Pneumonia severity index
SBP: Systolic blood pressure
SIRS: Systemic inflammatory response syndrome
UHPLC: Ultra-high-performance liquid chromatography
