# Supplementary material for: Time-dependent association of glucocorticoids with adverse outcome in community-acquired pneumonia: a 6-year prospective cohort study
Source: Crit Care. 2017 Mar 24;21:72. doi: 10.1186/s13054-017-1656-7 (PMC5364618; doi:10.1186/s13054-017-1656-7)
Supplement: Additional file 1: Table S1. — Baseline characteristics overall and stratified by 30-day adverse outcome, including the combined endpoint death/ICU admission in CAP. Data are presented as median [IQR] or number (percent); p values are considered statistically significant at p < 0.05. Bold values indicate statistical significance. CAP, Community-acquired pneumonia; CRP, C-reactive protein; CURB65, Confusion of new onset, blood urea nitrogen >7 mmol/L, respiratory rate ≥30 breaths per minute, systolic blood pressure <90 mmHg or diastolic blood pressure ≤60, and age ≥65 years; ICU, Intensive care unit; PAOD, Peripheral arterial occlusive disease; PCT, Procalcitonin; PSI, Pneumonia severity index; SBP, Systolic blood pressure; SIRS, Systemic inflammatory response syndrome. *Comorbidities were identified on the basis of medical records or patient report. (DOCX 21 kb) [file 13054_2017_1656_MOESM1_ESM.docx]

**Additional file 1**

Table S1. Baseline characteristics overall and stratified by 30-day adverse outcome including the combined endpoint death/ICU admission in CAP

|  |  |  |  |  |
| --- | --- | --- | --- | --- |
|  |  | **30-day adverse outcome (death, ICU admission)** | | |
| Characteristics | **Entire cohort**  **(n = 285)** | **Survivors, no ICU**  **(n = 254)** | **Non-survivors / ICU**  **(n = 31)** | ***p* value** |
|  |  |  |  |  |
|  |  |  |  |  |
| *Demographic characteristics* |  |  |  |  |
| Age | 71 [57, 81] | 71 [57, 81] | 76 [66, 82] | 0.17 |
| Male | 172 (60.4%) | 151 (59.4%) | 21 (67.7%) | 0.37 |
| *CAP characteristics* |  |  |  |  |
| PSI class |  |  |  |  |
| I | 32 (11.2%) | 31 (12.2%) | 1 (3.2%) | 0.13 |
| II | 55 (19.3%) | 52 (20.5%) | 3 (9.7%) | 0.15 |
| III | 52 (18.2%) | 51 (20.1%) | 1 (3.2%) | **0.022** |
| IV | 104 (36.5%) | 88 (34.6%) | 16 (51.6%) | 0.064 |
| V | 42 (14.7%) | 32 (12.6%) | 10 (32.3%) | **0.004** |
| CURB65 score |  |  |  |  |
| 0 | 63 (22.1%) | 61 (24.0%) | 2 (6.5%) | **0.026** |
| I | 67 (23.5%) | 63 (24.8%) | 4 (12.9%) | 0.14 |
| II | 82 (28.8%) | 71 (28.0%) | 11 (35.5%) | 0.38 |
| III | 57 (20.0%) | 51 (20.1%) | 6 (19.4%) | 0.92 |
| IV/V | 16 (5.6%) | 8 (3.1%) | 8 (25.8%) | **<0.001** |
| *Comorbidities** |  |  |  |  |
| Coronary heart disease | 59 (20.7%) | 50 (19.7%) | 9 (29.0%) | 0.23 |
| Congestive heart failure | 44 (15.4%) | 39 (15.4%) | 5 (16.1%) | 0.91 |
| Cerebrovascular insult | 28 (9.8%) | 21 (8.3%) | 7 (22.6%) | **0.011** |
| PAOD | 17 (6.0%) | 13 (5.1%) | 4 (12.9%) | 0.084 |
| Chronic renal failure | 67 (23.5%) | 51 (20.1%) | 16 (51.6%) | **<0.001** |
| Diabetes mellitus | 55 (19.3%) | 47 (18.5%) | 8 (25.8%) | 0.33 |
| Neoplastic disease | 38 (13.3%) | 32 (12.6%) | 6 (19.4%) | 0.30 |
| *Clinical history* |  |  |  |  |
| Fever | 185 (65.1%) | 165 (65.2%) | 20 (64.5%) | 0.94 |
| Chills | 87 (34.0%) | 79 (34.6%) | 8 (28.6%) | 0.52 |
| Glucocorticoid pretreatment | 22 (7.9%) | 21 (8.4%) | 1 (3.3%) | 0.33 |
| *Clinical findings* |  |  |  |  |
| Confusion | 20 (7.9%) | 12 (5.3%) | 8 (28.6%) | **<0.001** |
| Body temperature, °C | 38 [37.2, 38.8] | 38.0 [37.2, 38.8] | 37.9 [36.8, 38.5] | 0.17 |
| Breath rate, beaths/min. | 20 [16, 25] | 20 [16, 25] | 25 [20, 30] | **0.002** |
| Heart rate, beats/min. | 94 [82, 105] | 94 [82, 105] | 97 [80, 108] | 0.90 |
| SBP, mmHg | 130 [117, 148] | 130 [119, 148] | 129 [100, 150] | 0.22 |
| Arterial pH | 7.46 [7.42, 7.49] | 7.46 [7.43, 7.50] | 7.44 [7.35, 7.47] | **0.011** |
| SIRS criteria | 188 (66.0%) | 163 (64.2%) | 25 (80.6%) | 0.068 |
| *Outcome parameters* |  |  |  |  |
| Mechanical ventilation | 7 (2.5%) | 0 (0.0%) | 7 (22.6%) | **<0.001** |
| Septic shock | 6 (2.1%) | 0 (0.0%) | 6 (19.4%) | **<0.001** |
| Length of stay, days | 8 [5, 12] | 7.5 [5, 11] | 11 [7, 19] | **0.012** |
| *Admission laboratory findings* |  |  |  |  |
| CRP, mg/l | 132 [65, 252] | 128 [63, 247] | 159 [68, 358] | 0.15 |
| PCT, mcg/l | 0.48 [0.16, 3.20] | 0.45 [0.16, 3.12] | 0.53 [0.21, 6.30] | 0.27 |
| Cortisol, nmol/l | 402 [203.8, 723.2] | 394 [187.1, 721.1] | 454 [290.6, 1056.2] | 0.057 |
| 11-Deoxycortisol, nmol/l | 0.6 [0.17, 2.23] | 0.5 [0.17, 2.13] | 0.8 [0.18, 2.32] | 0.59 |
| Cortisone, nmol/l | 32.5 [18.42, 46.72] | 32.4 [17.93, 47.71] | 32.8 [21.44, 43.86] | 0.63 |
| Corticosterone, nmol/l | 8.7 [2.81, 25.02] | 8.6 [2.55, 24.35] | 8.7 [4.45, 28.38] | 0.26 |

*Data are presented as median [IQR] or number (percentage); p values are considered statistically significant at p<0.05. Bold values indicate statistical significance. CAP, community-acquired pneumonia; CRP, C-reactive protein; CURB65, ’Confusion’ ‘Urea’ ‘Respiratory rate’ ‘Blood pressure’ ‘Age >65 years’; ICU, intensive care unit; IQR, interquartile range; PAOD, peripheral artery occlusive disease; PCT, procalcitonin; PSI, pneumonia severity index; SBP, systolic blood pressure; SIRS, systemic inflammatory response syndrome.
*Comorbidities were identified based on medical records or patient report.*
